# The role of chikungunya virus capsid-viral RNA interactions in programmed ribosomal frameshifting

**DOI:** 10.1128/jvi.01393-25

**Published:** 2025-10-13

**Authors:** Jordan A. Farrington, Erin E. Rooney, Richard W. Hardy

**Affiliations:** 1 Department of Biology, Indiana University 1772, Bloomington, Indiana, USA; University of North Carolina at Chapel Hill, Chapel Hill, North Carolina, USA

**Keywords:** capsid, frame shifting, translation

## Abstract

Chikungunya virus (CHIKV) is a globally significant arthropod-borne virus that causes outbreaks in tropical and subtropical regions and is rapidly expanding in geographical range. Infection can lead to acute febrile illness with debilitating joint pain that may persist as chronic arthritis. Efficient CHIKV replication and virion assembly rely on coordinated interactions between viral RNA and the capsid protein, a structural component essential for particle formation. In addition to its well-established structural role, the alphavirus capsid protein has been shown in Sindbis virus to bind viral RNA and stabilize the genome, while in New World alphaviruses, it contributes to immune evasion through inhibition of host gene expression. However, its potential regulatory functions in viral RNA processes remain less understood. Here, we show that CHIKV capsid modulates programmed ribosomal frameshifting (PRF) within the 6K/Transframe (TF) region, influencing the balance between structural protein synthesis and TF production. Using a previously engineered CHIKV mutant (9900), which contains silent mutations that reduce capsid binding within 6K/TF, we demonstrate that diminished capsid-vRNA interaction correlates with increased PRF efficiency. This is associated with enhanced viral replication in immune-competent cells, yet this enhanced replication is not further improved by JAK-STAT inhibition, indicating that capsid binding already modulates immune response pathways. These findings identify CHIKV capsid as a potential modulator of PRF, expanding our understanding of alphavirus gene expression and RNA-protein interactions.

**IMPORTANCE:**

The multifunctional roles of the chikungunya virus (CHIKV) capsid protein—particularly its RNA-binding properties and potential to influence translation—represent important aspects of viral replication and pathogenesis. This study focuses on the CHIKV capsid’s influence on PRF, a key step in viral protein synthesis. Similar capsid-vRNA binding regions have been described in other alphaviruses, such as Venezuelan equine encephalitis virus (B. D. Carey, I. Akhrymuk, B. Dahal, C. L. Pinkham, et al., PLOS Pathogens 16:e1008282, 2020, https://doi.org/10.1371/journal.ppat.1008282), suggesting that capsid-mediated modulation of translation may represent a partially conserved mechanism across the alphavirus genus. By elucidating a role for capsid in PRF modulation, this study highlights a previously unrecognized layer of gene regulation in alphaviruses with broad implications for immune evasion, viral fitness, and translational control.

## INTRODUCTION

Chikungunya virus (CHIKV) is an Old-World alphavirus that poses a significant global health concern due to its potential to cause widespread outbreaks and severe disease. The virus is prevalent in tropical and subtropical regions and is primarily transmitted through the bites of infected *Aedes* mosquitoes, most commonly *Aedes aegypti* and *Aedes albopictus* (1, 2). CHIKV causes chikungunya fever, a disease characterized by fever, rash, and debilitating joint pain that can persist for months to years (3, 4). As global temperatures rise and *Aedes* mosquito habitation zones broaden, the risk of CHIKV outbreaks in naïve populations increases, underscoring the need for continued surveillance and research (5–7).

CHIKV has a single-stranded, positive-sense RNA genome with two large open reading frames (ORFs). The nonstructural ORF encodes replication proteins, while the structural ORF—expressed from a subgenomic RNA (sgRNA)—produces proteins essential for virion assembly (8–10). A key structural component is the capsid protein, derived from the sgRNA, which packages the viral genomic RNA into nucleocapsid cores (11–15). Beyond its structural role, the alphavirus capsid protein is a well-documented RNA-binding protein, as seen in Sindbis virus (SINV) and Venezuelan equine encephalitis virus (VEEV) (16, 17). Its interaction with viral RNA (vRNA) contributes to genome stability and, in combination with host proteins, can modulate infection dynamics (16, 17). With the increasing use of advanced RNA-binding analyses such as cross-linked immunoprecipitation and high-throughput sequencing (CLIP-Seq), cross-link-assisted mRNP purification, and RNA-interactome capture, the repertoire of host and viral RNAs interacting with alphavirus capsid proteins is expected to expand (18–20).

Building on these insights, our previous work mapped CHIKV capsid protein associations to the vRNA and explored the functional consequences of disrupting these interactions (21). Using CLIP-Seq, we identified a strong interaction between the CHIKV capsid protein and the 6K/Transframe (TF) coding region of the vRNA (21). However, the specific functional impact of capsid binding to this region remained unclear. Notably, this binding site overlaps a regulatory region involved in programmed ribosomal frameshifting (PRF), a mechanism that finely regulates protein synthesis by enabling ribosomes to shift reading frames with temporal and stoichiometric precision (22, 23).

In alphaviruses, the canonical polyprotein derived from the sgRNA includes 6K and the downstream glycoprotein E1 (8). However, when −1 PRF occurs at the 6K/TF junction, translation of 6K and E1 is reduced in favor of TF production (23, 24). TF, a viroporin, enhances viral replication and virion maturation while also interfering with the host interferon (IFN) response through mechanisms that remain incompletely understood (25–27). While neither 6K nor TF is strictly required for viral proliferation, both contribute to efficient viral propagation (28, 29).

Given the critical role of PRF in regulating the balance between 6K and TF synthesis, we hypothesized that capsid binding to the 6K/TF region may influence PRF efficiency and, in turn, impact CHIKV replication. While CHIKV capsid is well known for its structural and RNA-binding roles, its potential to modulate translational processes like PRF has not been explored. Here, we combine mutational analysis, dual-luciferase assays, and infection studies in immune-competent cells to investigate this interaction. These experiments reveal a previously unrecognized function for capsid in influencing PRF and offer insight into how alphaviruses may fine-tune gene expression in response to host immune pressure.

## RESULTS

### Capsid binding**,** not RNA sequence alone, modulates frameshifting 

**Fig 1:**
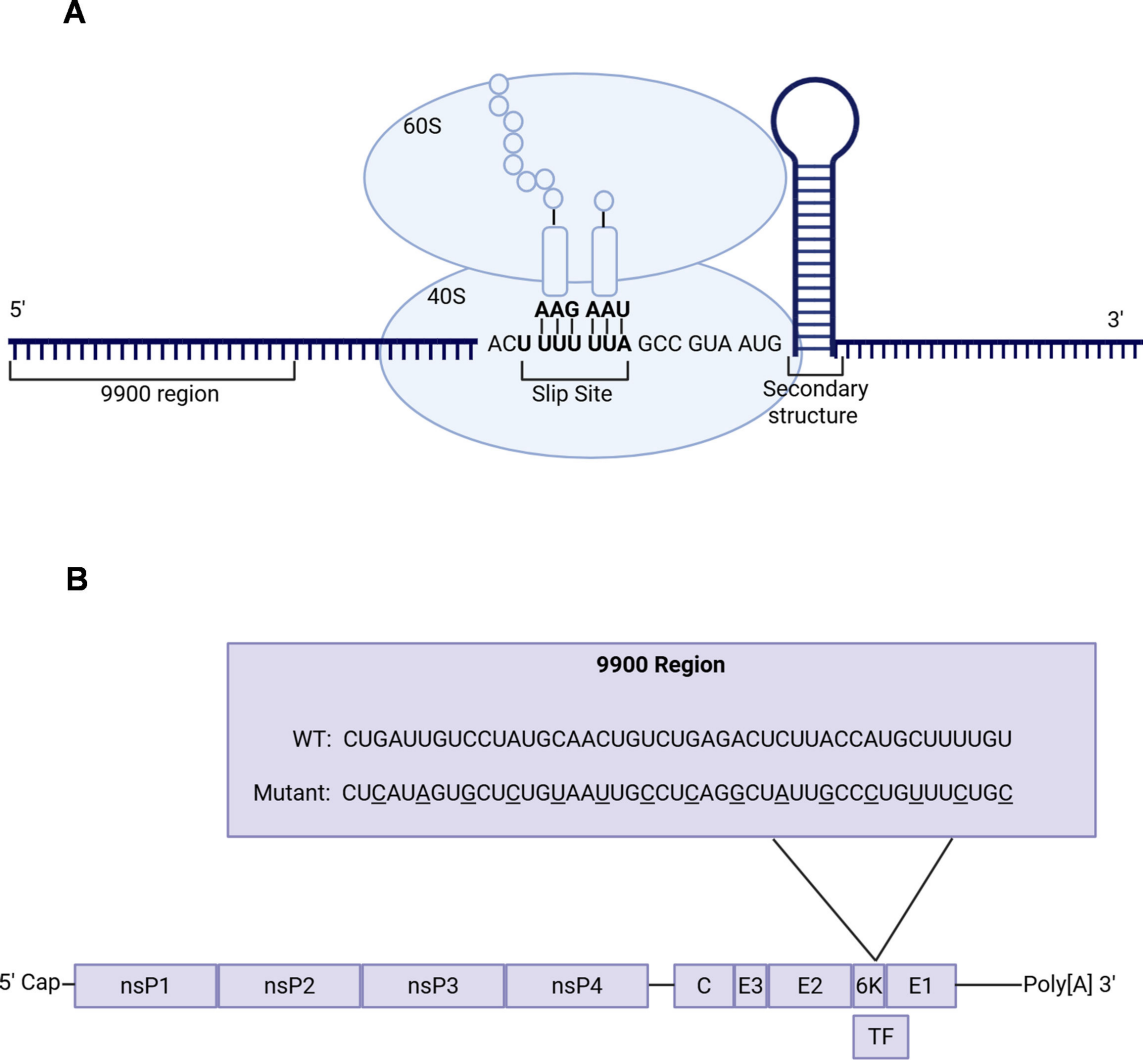
The 9900 region is 12 bases upstream of the minimal frameshifting elements. (**A**) A schematic representation of the proximity of the 9900 region to the PRF signals, notably the slippery site and secondary RNA structure in the canonical reading frame. The actively translating ribosome is represented as the 60S and 40S ribosomal subunits, where the machinery becomes paused on the slip site as the secondary structure is unwound. (**B**) Schematic of the CHIKV181/clone 25 genome, highlighting the wild-type 9900 region (nucleotides 9869–9913) and corresponding silent mutations introduced to create the 9900 mutant.

We previously mapped a prominent CHIKV capsid binding site to the 9900 region, which is located 12 bases upstream of a site containing essential elements for PRF in the 6K/TF coding sequence (Fig. 1A) (21). PRF is a mechanism that enables a nonrandom shift of the ribosome into alternative reading frames, mediated by two critical elements: a heptanucleotide sequence (slip site) and a downstream RNA secondary structure (Fig. 1A) (30).

Translation of alphavirus sgRNA produces a polyprotein that is processed into the capsid, E3, E2, 6K, and E1 structural proteins (Fig. 1B) (23). However, when a frameshifting event occurs, translation results in the production of a polyprotein processed into capsid, E3, E2, and TF proteins instead (Fig. 1B) (23). During frameshifting, the ribosome pauses on the slip site as it unwinds the downstream RNA structure. It then shifts back by one nucleotide, entering the −1 reading frame and accessing the TF coding sequence (30). Given the proximity of the 9900 region to the PRF signals in the 6K/TF coding region, we hypothesized that capsid binding might influence frameshifting events. To disrupt the capsid-vRNA interaction, we used the 9900 mutant sequence, which was engineered with silent mutations in the 6K/TF region to weaken this interaction, as previously shown by quantitative immunoprecipitation (qIP) following viral infection (Fig. 1B) (21).

**Fig 2:**
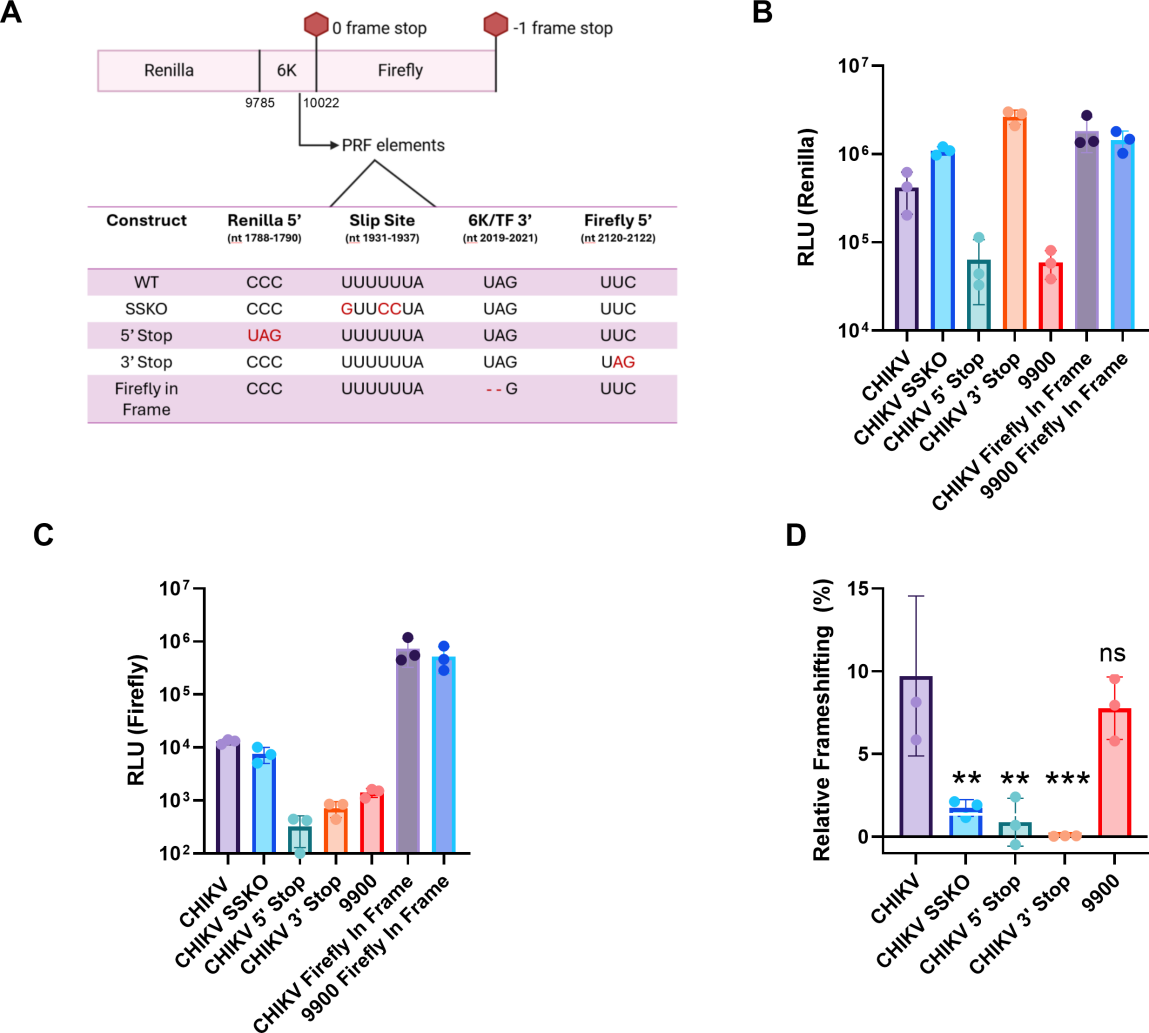
Wild-type (WT) and 9900 undergo −1 PRF similarly in an *in vitro* translational system absence of viral proteins. (**A**) Schematic of the dual-luciferase reporter system used to evaluate −1 PRF at the CHIKV 6K/TF region (CHIKV 181/clone 25 nucleotides 9,785–10,022). Constructs include the WT sequence, slippery sequence knockout (SSKO), and mutations introducing stop codons (5′ stop and 3′ stop) to assess PRF. The firefly in-frame control was included that mutates the 0-frame stop codon to allow translation of the downstream firefly luciferase. Mutated residues are shown in red. Nucleotide positions of each mutation in the table are indicated relative to the plasmid backbone. (**B**) Renilla luciferase activity (RLU, relative light units) serves as a translation efficiency control for constructs in a rabbit reticulocyte translation system. Mock, representing no-transfection control, showed low background Renilla activity (~10^4^ RLU). (**C**) Firefly luciferase activity (RLU), where increased firefly luciferase activity indicates frameshifted translation events. Mock displayed minimal background Firefly activity (~10^2^ RLU). (**D**) Frameshifting efficiency was calculated as: [(firefly RLU – mock firefly RLU)/(renilla RLU – mock renilla RLU)], then normalized to the ratio obtained from a Firefly In-Frame (FF IF) positive control in panel **A***,* which was set to 100% frameshifting efficiency. CHIKV constructs were compared to the CHIKV FF IF control, and 9900 constructs were compared to the 9900 FF IF control. Statistical significance was assessed by one-way ANOVA with Tukey’s post hoc test to correct for multiple comparisons relative to WT (**P < 0.01; ***P < 0.001; ns = not significant). Data are shown as mean ± SEM of biological triplicates.

To investigate frameshifting, we leveraged a dual-luciferase plasmid system to quantify frameshift efficiency across various synthetic DNA constructs (31, 32). In this system, the CHIKV 6K/TF sequence (nucleotides 9785-10022 from CHIKV strain 181/clone 25) is inserted between the zero-frame renilla luciferase and −1-frame firefly luciferase coding sequences (Fig. 2A). The renilla luciferase serves as a control for translation efficiency, while the firefly luciferase acts as a reporter for TF synthesis, which occurs when the ribosome shifts into the −1 reading frame. This design allows us to measure the ratio of firefly to renilla luciferase activity to determine frameshift efficiency. To prevent interference with luciferase activity and ensure proper separation of the two reporter proteins, foot-and-mouth disease virus 2A peptide sequences were placed after the renilla luciferase and test insert (Fig. 2A). This design minimizes potential effects of the test sequence on enzymatic function, ensuring consistent comparison between the in-frame control (explained below) and experimental constructs (33, 34).

Control constructs include the slip site knockout (SSKO), which prevents slippage at the heptanucleotide sequence; a 5′ stop codon to test for readthrough to the −1 frame encoding firefly luciferase; and a 3′ stop codon to evaluate for cryptic splice sites introduced by the test sequence (Fig. 2A) (35). In addition, an in-frame firefly luciferase control was generated for each test sequence. In this control, the firefly luciferase coding sequence was placed directly downstream of renilla luciferase and the inserted 6K/TF sequence, maintaining the 0-reading frame throughout (Fig. 2A) (35). This configuration allows uninterrupted translation of both luciferases and produces the maximal firefly signal expected in the absence of a frameshift event. Firefly activity from these in-frame constructs was used to normalize the firefly signal from experimental constructs, enabling accurate calculation of frameshift efficiency. Frameshifting efficiency was calculated using the formula:


$$\left(\frac{\text{Firefly RLU}-\text{Mock Firefly RLU}}{\text{Renilla RLU}-\text{Mock Renilla RLU}}\right)\times 100=\%\;\text{PRF},$$

and then expressed relative to the corresponding in-frame firefly control (35). Together, these controls ensured that the observed frameshift is due to the 6K/TF sequence itself rather than artifacts of the assay system.

Given the sensitivity of PRF to RNA structure and potential interactions with host or viral factors, we first evaluated our wild type (WT), 9900, and related control constructs in a minimal, cell-free translation system using rabbit reticulocyte lysates (RRL). This approach not only allowed us to study PRF in the absence of viral proteins but also provided a controlled context, reducing the impact of host interactions. Since the 9900 mutations could conceivably influence RNA structure or host protein interactions, isolating their direct impact on translation in this simplified system was essential.

Renilla luciferase, serving as the translation control, consistently exhibited levels at least 10-fold higher than background, confirming translation of all constructs (Fig. 2B; Fig. S1A). Firefly luciferase, representing the frameshift product and serving as a proxy for TF synthesis, showed comparable levels between CHIKV and 9900 constructs (Fig. 2C; Fig. S1B). Analysis of frameshifting efficiency indicated that, in this system, frameshifting occurred in 7%–9.5% of translational events for both the WT and 9900 constructs with no statistically significant difference in frameshifting between the two sequences (Fig. 2D; Fig. S1C). In all cases, frameshifting frequency on control RNAs was significantly lower (0%–1.2%).

### CHIKV capsid binds the 6K/TF region in reporter constructs.

**Fig 3:**
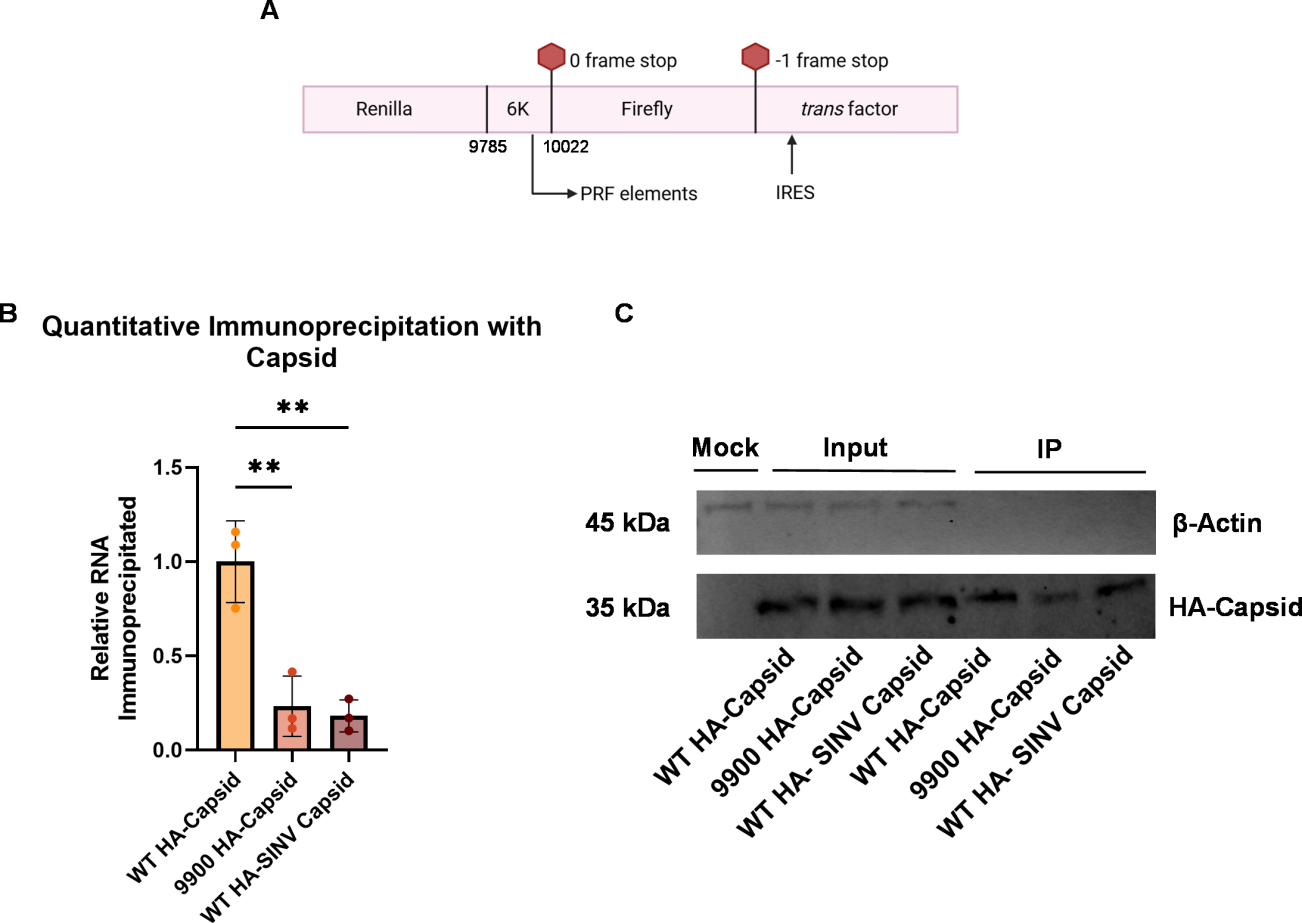
qIP demonstrates capsid binding to the WT dual luciferase construct, but not to the 9900 mutant. (**A**) A diagram of the dual-luciferase reporter construct is shown, featuring renilla luciferase, the 6K/TF gene (CHIKV 181/clone 25 nt 9785–10022), and firefly luciferase. The stop codons represent the native 0-frame termination site, while the −1-frame stop codon marks the end of the firefly sequence translated via −1 PRF. An IRES element is located downstream of the frameshifting cassette, followed by either CHIKV or SINV HA-tagged capsid (*trans* factor). (**B**) qRT-PCR analysis after qIP of the 6K/TF region inserted into the dual luciferase reporter. The IP targeting the CHIKV (HA-Capsid) or SINV capsid (HA-SINV Capsid) protein reveals significantly higher co-immunoprecipitation of RNA with the WT HA-capsid construct compared to the 9900 HA-capsid mutant and WT HA-SINV capsid construct. RNA levels were normalized to GAPDH and are presented relative to WT HA-capsid. Statistical significance was determined using an ordinary one-way ANOVA (**P < 0.01). Data represent the mean ± SD of biological triplicates. (**C**) Western blot analysis of HA-tagged capsid protein before and after the qIP process in mock (no plasmid transfected), WT, 9900, and SINV capsid dual luciferase construct transfected samples. β-Actin acts as the cellular control. All western blot experiments were repeated at least three times independently with similar results. Representative images are shown.

While the RRL system provided a controlled environment to assess intrinsic frameshifting efficiency, it lacked the viral and cellular factors that could influence PRF modulation in a biological context. To explore capsid’s role in modulating PRF in CHIKV, we incorporated it into the dual-luciferase system to evaluate its impact on frameshifting efficiency. CHIKV capsid expression was driven by an encephalomyocarditis virus (EMCV)-derived internal ribosome entry site (IRES) element positioned downstream of the frameshifting cassette to enable translation of capsid without dependence on upstream frame continuity. Since the antibody we had for capsid showed significant non-specific interactions (not shown), the capsid protein expressed from our construct was tagged with a hemagglutinin (HA) epitope at its N-terminus, allowing clean detection (“*trans* factor,” Fig. 3A) (36).

To confirm capsid’s binding to the 6K/TF region of the reporter constructs and to distinguish this from capsid’s known tendency for non-specific RNA interactions, we performed qIP in HEK293T cells transfected with the dual-luciferase constructs. RNA recovery from the IP was quantified and normalized for both WT HA-capsid (representing capsid binding to the RNA) and 9900 HA-capsid (which has disrupted capsid binding due to silent mutations). The retention of the capsid-RNA interaction was significantly reduced (approximately fourfold) in the 9900 construct compared to the WT, confirming that WT capsid efficiently binds to the RNA derived from the dual luciferase constructs, whereas the mutations in the 9900 construct impair this interaction (Fig. 3B).

To further validate these findings, we replaced the *trans* factor by expressing the SINV capsid protein, rather than CHIKV capsid (Fig. 3A). Consistent with the earlier results, SINV capsid did not efficiently bind the WT 9900 region as well as the CHIKV capsid (Fig. 3B), mirroring the reduced interaction seen with CHIKV 9900 HA-capsid and supporting the specificity of the capsid-RNA interaction.

Importantly, the levels of capsid protein pulled down in these experiments were similar for each construct (Fig. 3C), indicating that the difference in RNA binding was not due to differential protein expression but rather due to the disrupted binding capability of the capsid protein. This result supports our earlier findings, where we observed reduced RNA recovery following capsid IP in the 9900 virus compared to the WT virus in a full viral context (21). These data support CHIKV capsid binding to RNA containing the 6K/TF region, consistent with prior mapping (21), and the 9900 mutations effectively disrupt this interaction without altering capsid protein expression.

### Capsid suppresses −1 PRF via binding to the 6K/TF region

The binding of the capsid protein to our dual luciferase constructs confirms its interaction with CHIKV dual luciferase-derived RNA specifically, establishing a foundation for further investigation into its modulatory effects on PRF. This binding interaction provides a direct means to assess how capsid influences PRF efficiency within the context of our reporter system.

**Fig 4:**
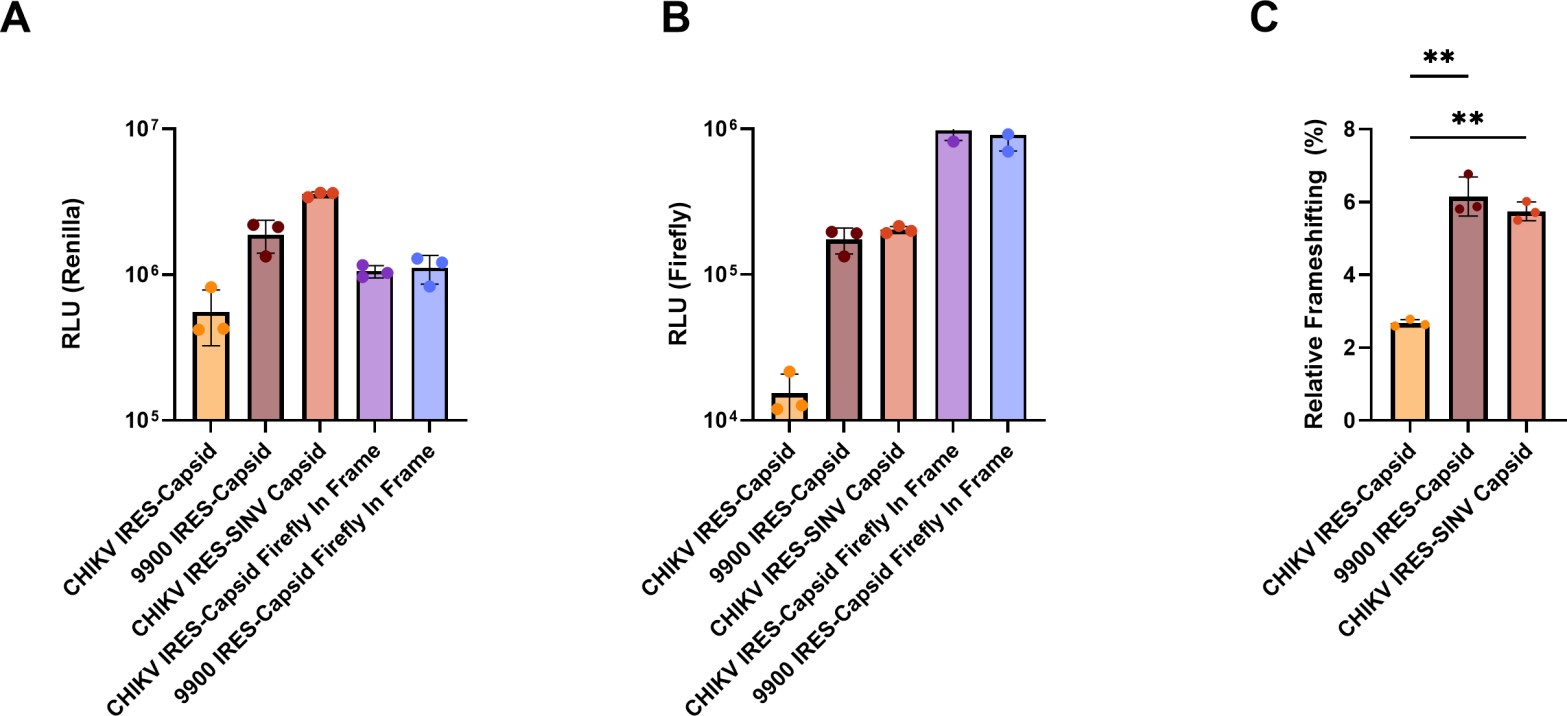
CHIKV capsid protein influences −1 PRF. (**A**) Renilla luciferase activity (RLU) is presented as a measure of translation occurring in the 0-frame. Renilla signal in the mock condition remained low (~10^4^ RLU), indicating minimal background translation. (**B**) Firefly luciferase activity (RLU) reflects translation via −1 PRF. Mock showed minimal firefly background (~10^3^ RLU). (**C**) Relative frameshifting efficiency was calculated as described and then normalized to the ratio obtained from a firefly in-frame positive control set as 100% frameshifting efficiency. CHIKV IRES-capsid and CHIKV IRES-SINV capsid were made relative to the CHIKV IRES-capsid firefly in-frame control, and 9900 IRES-capsid to 9900 IRES-capsid firefly in-frame control. Statistical significance was assessed by one-way ANOVA with Tukey’s post hoc test to correct for multiple comparisons relative to WT IRES-capsid (**P < 0.01). Data are shown as mean ± SEM of biological triplicates.

Following transfection of the capsid-expressing reporter constructs in HEK293T cells, renilla luciferase activity (reflecting 0-frame translation) was consistent across all constructs with at least a 100-fold increase compared to background readings (Fig. 4A; Fig. S2A), confirming RNA integrity and proper translation. Firefly luciferase activity, indicating frameshift product formation, revealed differences in alternative reading frame translation among the constructs (Fig. 4B; Fig. S2B). Frameshifting efficiency, calculated as the firefly-to-renilla ratio and normalized by the respective CHIKV capsid-containing in-frame control, was lowest for WT IRES-HA capsid (Fig. 4C; Fig. S2C-D) with an average of 2.67%. However, the 9900 IRES-HA capsid construct, which disrupts capsid binding through silent mutations in the RNA, showed significantly higher frameshifting efficiency (6.15%) on average compared to WT IRES-HA capsid. This suggests that the absence of capsid binding relieves suppression of PRF.

When SINV capsid, rather than CHIKV capsid, was present in the reporter system (Fig. 3A), frameshifting efficiency averaged 5.74% when normalized to the CHIKV firefly in-frame construct, statistically indistinguishable from that seen for 9900 with CHIKV capsid (Fig. 4C). These findings demonstrate that CHIKV capsid binding to the WT 9900 region could play a key role in restricting PRF. Replacing CHIKV capsid with non-binding SINV capsid (Fig. 3B) or introducing silent mutations in the 9900 region that reduce capsid-RNA interaction consistently increases −1 PRF efficiency at the 6K/TF site. These findings indicate that capsid binding can suppress frameshifting, highlighting a modulatory role for CHIKV capsid in fine-tuning viral protein synthesis and potentially shaping replication dynamics and immune evasion strategies.

To ensure that the increased frameshifting observed in the 9900 construct was not due to changes in transcript abundance, we performed absolute quantification of renilla and firefly RNA levels by qRT-PCR using standard curves. RNA expression was equivalent between the CHIKV and 9900 dual luciferase constructs (Fig. S3). This confirms that changes in PRF efficiency reflect translational modulation rather than differences in transcript abundance.

### Disruption of capsid binding enhances replication in immune-competent cells

**Fig 5:**
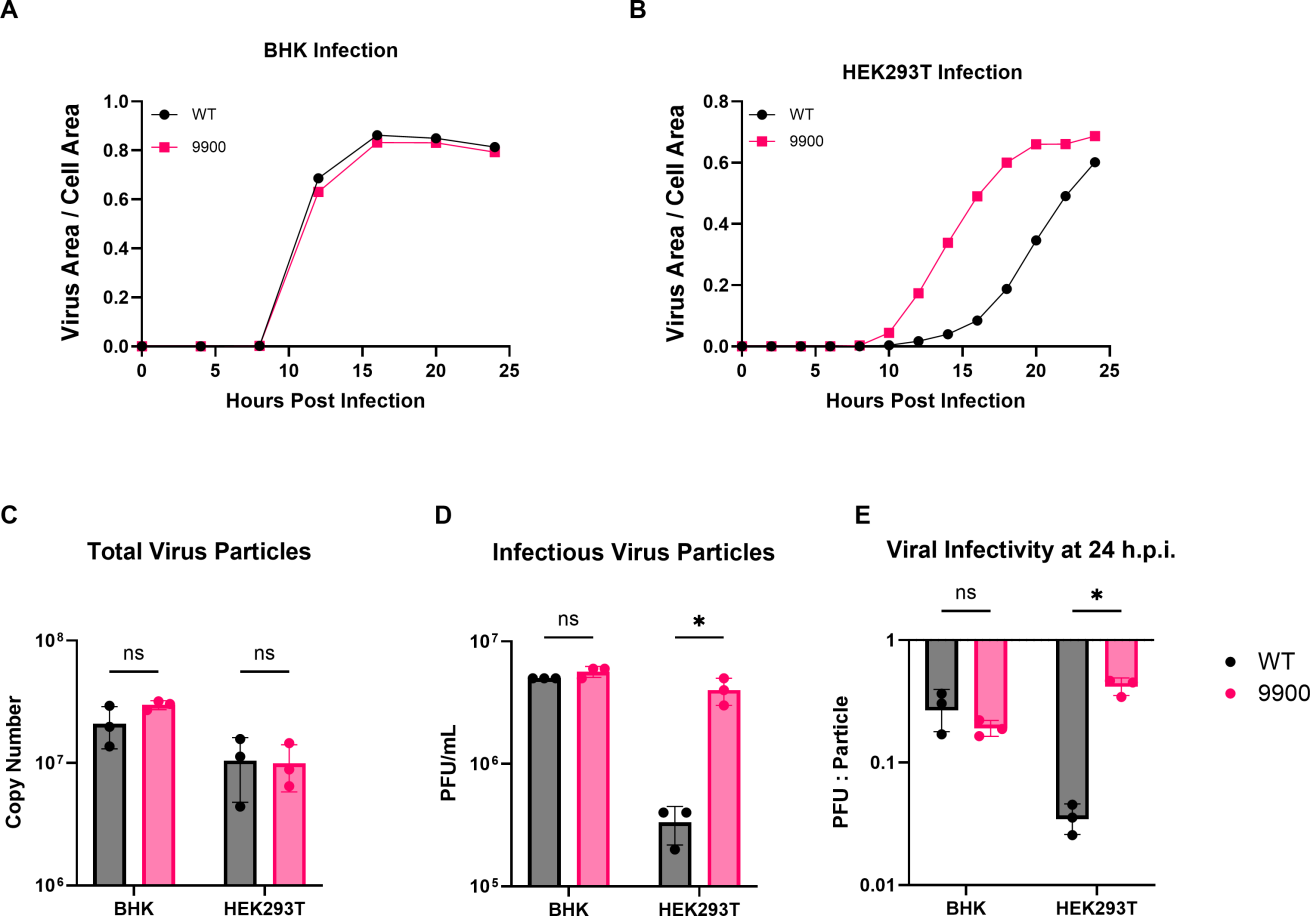
Disruption of capsid binding to the 9900 region enhances viral spread and specific infectivity in immune-competent cells. (**A and B**) BHK and HEK293T cells were infected at an MOI of 5 PFU/cell with WT or 9900 mKate-tagged CHIKV, in which a fluorescent reporter was inserted between the capsid and E3 genes. Infection progression was monitored by live-cell imaging of mKate fluorescence, used as a proxy for viral area. Data were normalized to phase-contrast measurements of total cell area over time. (**C**) vRNA copy number in the supernatant was quantified by qRT-PCR at 24 hours post-infection using primers against the CHIKV nsP2 region and normalized to a standard curve. (**D**) Infectious viral titers were determined from the same supernatants by plaque assay on BHK cells and reported as PFU per milliliter. (**E**) Specific infectivity was calculated as the ratio of infectious particles (PFU) to total viral genomes, with data log10-transformed. All data represent the mean of three biological replicates. Statistical significance was determined by multiple paired *t*-tests with Bonferroni correction (*P < 0.05; ns = not significant).

To assess the biological consequences of capsid-mediated PRF influence, we next examined viral replication kinetics of WT and 9900 CHIKV in both IFN-deficient and immune-competent cell types. To directly explore the consequences of capsid binding to the 6K/TF region in CHIKV and 9900 infectivity, we infected BHK cells with mKate-tagged WT and 9900 viruses at a high multiplicity of infection (5 PFU/cell). BHK cells are impaired in key antiviral pathways, namely type I interferon (37), which makes them an ideal model for studying viral replication and particle production without the influence of antagonistic host immune factors. We found that WT and 9900 viruses replicate with similar kinetics at a high multiplicity of infection (MOI), suggesting that the presence of the mutations does not significantly impact entry, replication efficiency, or assembly of virions compared to WT virus under these conditions (Fig. 5A).

After observing no notable differences in BHK cells, we chose to infect a cell line better equipped to mount an innate immune response in order to more accurately replicate a typical mammalian host environment. HEK293T cells were selected for this purpose due to their ability to initiate IFN-I signaling (38, 39). Cells were infected at 5 PFU/cell with the mKate-tagged CHIKV and 9900 viruses to investigate how immune defense barriers are navigated when overwhelmed, and the resulting effects on viral replication. Notably, the replication of the 9900 mutant was observed 4 hours earlier than the WT virus, reaching its peak within 24 hours, while the WT had not yet done so (Fig. 5B). This suggests that the mutant virus may overcome immune-mediated barriers more rapidly.

Expectedly, infection with both viruses in BHK cells resulted in a comparable number of infectious virions in the medium at 24 hours post-infection (HPI), as determined by qRT-PCR detection of viral genomes and plaque assay titration on BHK-21 cells (Fig. 5C through E). Interestingly, in HEK293T cells under the same conditions, the number of virions was similar for both viruses (Fig. 5C). However, the 9900 mutant achieved ~1.5 log_10_ higher titer (Fig. 5D), implying there were more infectious virions. As a result, the ratio of infectious particles to total particles increased from approximately 1:50 in WT infection to 1:5 in 9900 infection (Fig. 5E). This suggests that weakened capsid binding may play a critical role in infectivity in immunocompetent cells. These results suggest that enhanced TF synthesis may contribute to more efficient immune evasion or viral entry dynamics in HEK293T cells, potentially through elevated TF synthesis that improves immune evasion or alters viral entry dynamics.

### Ablation of PRF negates 9990 growth phenotype

To determine whether the observed phenotype depends on PRF-mediated TF production, we next generated slip site knockout versions of both the WT and 9900 viruses. The SSKO mutation consisted of silent changes to the heptanucleotide slippery sequence required for −1 PRF, thereby aiming to prevent ribosomal frameshifting without altering the downstream TF coding region (Fig. 2A) (35). This design was meant to maintain all other aspects of the genomes explored previously while eliminating TF expression.

The rationale for this approach was based on the proposed model in which reduced capsid binding in the 9900 mutant relieves WT levels of apparent suppression of PRF, thereby elevating TF levels and enhancing replication in the presence of an intact interferon response. If TF production is the key driver of the observed phenotype, abolishing PRF in the 9900 background should remove its replication advantage and yield kinetics comparable to WT SSKO virus.

**Fig 6:**
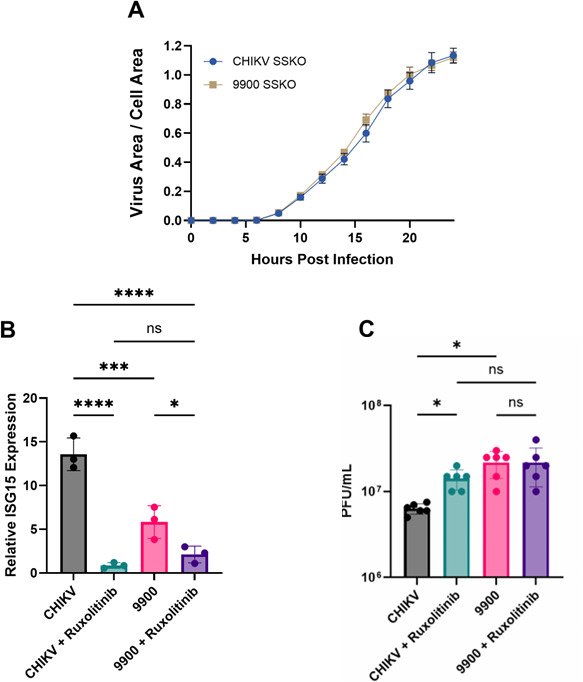
Ruxolitinib treatment increases WT CHIKV titers but does not affect 9900 mutant replication. (**A**) HEK293T cells were infected at an MOI of 5 PFU/cell with WT SSKO or 9900 SSKO mKate-tagged CHIKV. Infection progression was monitored by live-cell imaging of mKate fluorescence, used as a proxy for viral area. Data were normalized to phase-contrast measurements of total cell area over time. (**B**) Relative ISG15 expression in HEK293T cells infected for 48 hours with WT CHIKV or the 9900 mutant at 0.1 PFU/cell, with or without Ruxolitinib treatment. CHIKV infection induces ISG15 expression, which is significantly reduced upon JAK-STAT inhibition with Ruxolitinib. The 9900 mutant shows lower baseline ISG15 induction, with further reduction upon treatment. qRT-PCR was performed in biological triplicate and technical duplicate. (**C**) Viral titers (PFU/mL) of WT CHIKV and the 9900 mutant in the presence or absence of ruxolitinib. While WT CHIKV titers increase with ruxolitinib treatment, the 9900 mutant remains unaffected, suggesting that the mutation may already counteract the IFN response. Titers were determined from six biological replicates. Statistical significance was assessed by one-way ANOVA with Tukey’s post hoc test to correct for multiple comparisons. Data are presented as mean ± SEM, with statistical significance indicated (*P < 0.05, ***P < 0.01, and ****P < 0.001; ns = not significant).

Replication kinetics of the SSKO viruses were assessed in HEK293T cells at a high multiplicity of infection (5 PFU/cell). Both WT SSKO and 9900 SSKO viruses exhibited nearly identical replication curves, with similar onset times, slopes, and peak infection plateaus (Fig. 6A). Importantly, the accelerated spread seen for the 9900 mutant was absent in the SSKO background, confirming that the enhanced replication of 9900 requires an intact PRF site and is likely not attributable to other features of the 9900 mutations.

### Role for PRF in antagonizing interferon signaling

Given that the 9900-growth advantage is abolished when PRF is disrupted, we next tested whether its enhanced replication in immune-competent cells reflects resistance to type I IFN signaling. TF has been implicated as an antagonist of the IFN response in alphaviruses (27), and our model predicts that elevated TF production in the 9900 mutant could diminish the effectiveness of JAK–STAT-mediated antiviral pathways. To directly assess this, we pharmacologically inhibited the JAK–STAT pathway with ruxolitinib, a potent and selective kinase inhibitor that blocks STAT1 phosphorylation and downstream interferon stimulated gene (ISG) induction (40, 41).

HEK293T cells were pretreated with 300 nM Ruxolitinib or vehicle (0.1% dimethyl sulfoxide [DMSO]) for 24 h prior to infection with WT CHIKV or the 9900 mutant (MOI 0.1 PFU/cell). After adsorption, treatments were maintained for the duration of infection. At 48 HPI, total RNA was collected for qRT-PCR analysis of interferon pathway activity, and infectious virus titers were determined by plaque assay.

Consistent with prior observations, WT CHIKV infection alone induced robust expression of ISG15, a canonical ISG (Fig. 6B) (42). Interestingly, while the 9900 mutant also induced ISG15 expression, its induction was markedly lower than that of CHIKV (by ~2.5-fold) and was further diminished by ruxolitinib, confirming that both viruses were responsive to JAK-STAT inhibition at the transcript level (Fig. 6B). These findings suggest that the 9900 mutant partially circumvents IFN-mediated transcriptional activation even in the absence of pharmacological inhibition.

The impact on viral replication mirrored these transcriptional patterns. Inhibition of the JAK-STAT pathway increased CHIKV infectious titers by twofold, consistent with the role of interferon signaling in restricting viral replication (Fig. 6C) (42). In contrast, ruxolitinib had no detectable effect on replication of the 9900 mutant, which remained high regardless of treatment (Fig. 6C). These results suggest that the 9900 mutant replicates efficiently despite intact IFN signaling. While TF expression is a potential contributor to this effect, how TF antagonizes the IFN response remains unclear. Elucidating this mechanism in future work would allow us to build directly on the findings presented here.

## DISCUSSION

In this study, we demonstrate that the CHIKV capsid protein modulates programmed ribosomal frameshifting at the 6K/TF region of the viral RNA by binding near the −1 PRF site. Disrupting this interaction through silent mutations in the 9900 region increases frameshifting efficiency, suggesting enhanced TF production, and confers a replication advantage in immune-competent cells. As we were unable to reliably detect TF using available antibodies, we used dual-luciferase reporters to estimate TF synthesis via frameshifting efficiency. These findings establish a novel post-transcriptional role for capsid in modulating the balance of structural and accessory protein expression during CHIKV infection.

### Capsid binding suppresses PRF at the 6K/TF site

**Fig 7:**
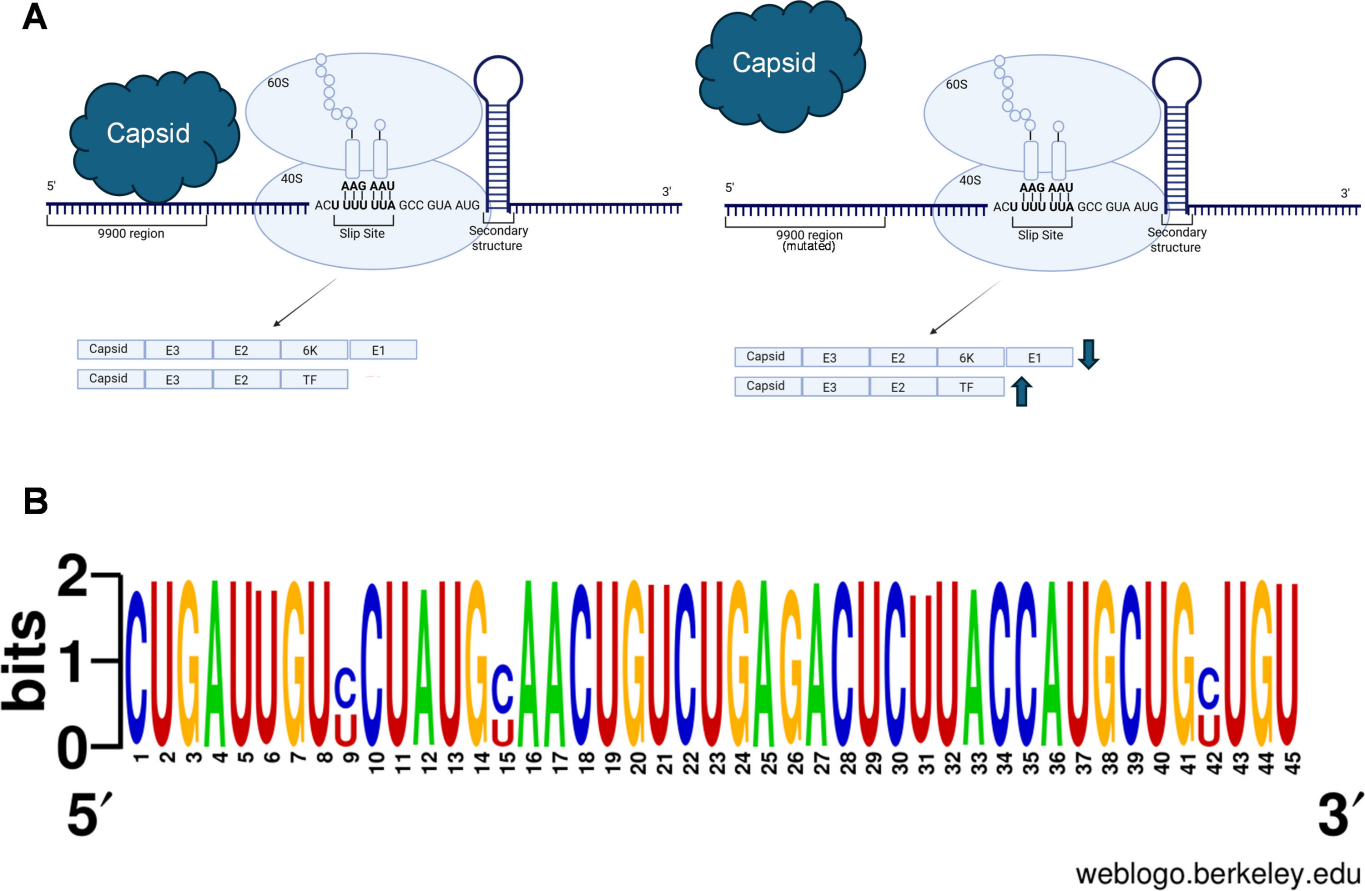
Proposed mechanistic model of capsid’s regulation on PRF. (**A**) Schematic illustrating how CHIKV capsid may modulate PRF in the context of wild-type and mutated 9900 viruses. Left: The wild-type primarily produces the canonical polyprotein (Capsid, E3, E2, 6K, and E1) with a higher efficiency, while only a small % undergoes frameshifting to produce an alternative product (Capsid, E3, E2, and TF). Right: a mutation in the 9900 region reduces the efficiency of the canonical polyprotein production, increasing frameshifting. Although the capsid is shown here directly binding the 9900 region, it is plausible that another host or viral factor is involved in this interaction. (**B**) Multiple Sequence Alignment of the 9900 region across all complete CHIKV genomes (NCBI Virus database, taxID: 37124), visualized using WebLogo (45). Conserved nucleotides are indicated by letter height. Underlined codons represent positions where naturally occurring variants match the mutations introduced in this study.

A major conclusion of this work is that CHIKV capsid partially suppresses −1 PRF at the 6K/TF junction by binding to an upstream vRNA sequence (Fig. 7A, left panel). This capsid-mediated interaction is suggested here to reduce ribosomal slippage and TF synthesis, as evidenced by the increased frameshifting efficiency observed in the 9900 mutant (Fig. 7A, right panel). Our dual-luciferase reporter data show that WT capsid reduces PRF frequency compared to both the binding-deficient 9900 mutant and SINV capsid, supporting a model in which capsid-vRNA binding contributes to PRF modulation in a virus-specific manner. Even modest changes in PRF (e.g., from ~3% to ~6%) can lead to significant differences in TF abundance, especially in the context of exponential translation amplification. As seen in other viruses, including HIV and coronaviruses (43, 44), such differences can meaningfully affect host response modulation and viral fitness.

This adds to growing evidence that alphavirus capsid proteins possess regulatory RNA-binding functions beyond structural roles, as has been observed in SINV and VEEV, where capsid binding to vRNA contributes to genome stability and translational control (16, 17). Notably, a VEEV capsid-binding site has been identified in a comparable region of the genome (17), suggesting that this mode of translational modulation may be conserved across certain alphaviruses.

### Impact on viral replication and immune evasion

The increase in PRF and the inferred elevation in TF synthesis in the 9900 mutant correlates with enhanced replication in immune-competent cells. This is likely due to TF’s known role in antagonizing type I IFN responses (27). Supporting this, the replication advantage of the 9900 mutant is lost in IFN-deficient BHK cells, and JAK-STAT inhibition boosts WT CHIKV replication but has no effect on the 9900 virus. Additionally, SSKO viruses, which aim to eliminate TF expression, replicate similarly in both WT and 9900 backgrounds, suggesting that the 9900 mutant’s advantage depends on functional TF production. These findings imply that enhanced TF expression in the 9900 mutant interferes with the impact of interferon signaling, allowing replication to proceed even in the presence of intact immune responses.

Importantly, these findings do not suggest that the WT virus is inherently less fit in natural settings; rather, they support a model in which CHIKV finely tunes PRF through capsid-RNA interactions to balance immune evasion with structural protein synthesis. Excessive TF production at the expense of 6K/E1 may disrupt glycoprotein stoichiometry or impair virion assembly, although direct evidence for these effects remains to be established. An additional consideration is the dual-host lifecycle of CHIKV. It remains plausible that capsid-mediated PRF modulation plays a more prominent role in the mosquito host, where different selective pressures may favor tight regulation of TF expression—an aspect not captured in mammalian cell culture models.

### Hypothesized model for PRF modulation

The mechanism by which CHIKV capsid suppresses PRF remains to be fully defined. One possibility is that capsid binding alters or stabilizes local RNA secondary structures near the PRF site, preventing efficient ribosome pausing or back slipping. Alternatively, capsid binding could sterically block ribosome movement at or near the PRF site. The precise nucleotide footprint of the CHIKV capsid on RNA is not yet defined; however, similar alphaviruses like SINV and VEEV suggest that capsid-RNA interactions typically span ~20–40 nucleotides (16, 17)—a range that could plausibly impact PRF efficiency if positioned near the stimulatory signals. We speculate that the functional consequence of this suppression is not to completely abolish TF expression, but rather to modulate it over the course of infection. Early in infection, when capsid levels are low, PRF efficiency may be higher, allowing transient TF production that could help suppress innate immune responses. As capsid accumulates, increasing capsid–RNA interactions could progressively reduce PRF, preventing excessive or prolonged TF expression that might disrupt viral protein stoichiometry or interfere with particle assembly. In this way, capsid-mediated PRF suppression could act as a feedback mechanism to fine-tune TF levels in a temporally regulated manner.

A mechanistically similar example is seen in SARS-CoV, where an upstream attenuator stem-loop positioned before the PRF structure physically impedes ribosome movement and reduces back-slipping efficiency, thereby tuning frameshifting output (44, 46). Whether CHIKV capsid directly alters RNA folding or exerts steric hindrance as a protein-based attenuator remains an open question—one that could be addressed using SHAPE-MaP or ribosome profiling approaches in future studies.

### Perspective and broader implications

Although disrupting capsid binding increases PRF and enhances replication in immune-competent cells, the 9900 mutations are absent in circulating CHIKV isolates (Fig. 7B). Of note, we did not perform dN/dS or synonymous site conservation analysis and instead report that these mutations are not found in currently available sequences. This may indicate that CHIKV maintains a conserved balance between TF and 6K/E1 production to preserve glycoprotein stoichiometry (8). Alternatively, the absence of 9900 mutations could reflect host-specific or transmission-related constraints not captured in mammalian cell culture. Further studies in *Aedes* mosquito cell lines could begin to answer some of these questions.

The involvement of a structural protein like capsid in influencing PRF highlights an additional layer of post-transcriptional control during the viral replication cycle. It also raises the possibility that similar capsid-RNA interactions may occur in other alphaviruses, though further studies would be needed to determine their functional relevance.

### Conclusions

Our findings identify CHIKV capsid as a modulator of PRF at the 6K/TF region and demonstrate that disrupting this interaction likely enhances TF expression, replication, and some resistance to interferon signaling. This study broadens our understanding of alphavirus gene expression strategies and highlights capsid-vRNA interactions as potential regulatory checkpoints that balance immune evasion, structural protein production, and replication fitness.

## MATERIALS AND METHODS

### Cell culture

Baby hamster kidney fibroblasts (BHK-21) and modified human embryonic kidney (HEK293T) cells were grown at 37°C with 5% CO_2_ in 1× minimal essential medium with 10% heat-inactivated fetal bovine serum, 1% L-glutamine, 1% non-essential amino acids, and 1% antibiotic-antimycotic solution (all purchased from Corning, Corning, NY, USA).

### Viruses

Plasmids encoding CHIKV strain 181/clone 25 (GenBank accession: MW473668.1), tagged with a mKate fluorophore and FMV2 element between capsid and E3, with either the wild type or 9900 mutated sequences and the respective SSKO viruses, were linearized with *SacI* (NEB, Ipswich, MA, USA). The inserted sequence includes the capsid coding region, followed by three amino acids from E3 to allow for autoproteolytic cleavage (ensuring mKate is not fused to capsid), the mKate reporter, and the FMV2 element, before the remaining portion of the structural polyprotein (47). The linearized plasmids were used for *in vitro* transcription (IVT) using SP6 RNA polymerase (NEB, Ipswich, MA, USA). IVTs were transfected into confluent BHK-21 cells using the LTX transfection reagent following the manufacturer’s protocol (Invitrogen, Waltham, MA, USA) with Opti-MEM I reduced serum medium (Gibco, Waltham, MA, USA). Four hours after transfection, the medium was removed and replaced with fresh 1× MEM. Approximately 52 hours post-transfection, the supernatant was harvested and clarified by centrifugation at 7,000 × *g* for 5 minutes. Clarified viral supernatant was aliquoted and stored at −80°C. Viruses were titered by standard plaque assay on confluent BHK-21 cells.

### Viral infection

BHK-21 and HEK293T cells grew to ~70% confluency in 12-well plates (Greiner Bio-One CellStar), then the original medium was discarded and replaced with 200 µL fresh 1× MEM. Cells were infected in triplicate with virus at an MOI of 5 PFU/cell and rocked for 1 hour at room temperature to allow for viral adsorption. The supernatant containing unbound virus and medium was aspirated, the cells were washed with 1× phosphate-buffered saline (PBS), and fresh 1× MEM was then added. The plates were then incubated at 37°C as described below.

### Viral spread by live cell imaging

Viral spread was measured via live cell imaging using the IncuCyte Live-Cell Analysis System (Essen Biosciences, USA) set to 37°C with 5% CO_2_. Briefly, BHK-21 and HEK293T cells were infected as described above, and images were captured from nine separate fields of view at 2-hour intervals over 24 hours. The results reflect the mean values from three independent biological replicates. The viral area measured by fluorescence generated by the mKate reporter was adjusted based on cell area.

### Specific infectivity

BHK-21 or HEK293T cells were infected with the indicated viruses at an MOI of 5 PFU/cell and harvested 24 HPI. The supernatant was collected, clarified by centrifugation at 7,000 × *g* for 5 minutes, and either used immediately for cDNA synthesis or stored at 4°C for short-term use. To quantify total viral genomes, an equal volume of RNA was reverse transcribed into cDNA using MMuLV Reverse Transcriptase (NEB, Ipswich, MA, USA) with random hexamer primers (Integrated DNA Technologies, Coralville, IA, USA). A standard curve was generated from serial dilutions of the CHIKV 181/25 plasmid. Quantitative RT-PCR was conducted with the SensiFAST SYBR Hi-ROX Master Mix (Meridian Bioscience, Memphis, TN, USA) and primers specific to the CHIKV nsP2 coding region, following the manufacturer’s protocol. Gene expression was measured using the Applied Biosystems StepOnePlus qRT-PCR system (Life Technologies, Carlsbad, CA, USA), with duplicate samples for both the standard curve and the experimental samples collected in biological triplicates. The Ct values from the standard curve were used to calculate the viral genomic copies in the samples. The infectious viral titer measured by standard plaque assay in BHK-21 cells was divided by the total genome copies to determine the PFU-to-particle ratio.

### Plasmid construction

Synthetic oligonucleotides were designed using SnapGene software (snapgene.com). The dual-luciferase plasmid backbone, pJD2257 (32, 48), was used for all constructs adjusted to prevent unintended splicing errors. The 6K/TF region of the CHIKV 181/25 genome (nt 9785–10022) with either the wild-type or mutant 9900 sequence was inserted between the renilla and firefly luciferase genes by PCR amplification with Q5 DNA polymerase (NEB, Ipswich, MA, USA), following the manufacturer’s protocol. Amplified DNA was digested with *DpnI* (NEB) and purified using the DNA Clean and Concentrator-5 kit (Zymo Research, Irvine, CA, USA). Purified DNA was eluted in 10 µL of nuclease-free H_2_O and ligated with NEBuilder (NEB). The ligated products were transformed into in-house DH5α competent cells and grown on LB agar plates with ampicillin. Colonies were screened via PCR using Taq polymerase (NEB) with primers specific to the insert. Positive colonies were confirmed by whole plasmid sequencing through Plasmidsaurus using Oxford Nanopore Technology and custom annotation. The same approach was used to insert an EMCV-derived IRES element downstream of the dual luciferase coding region, in order to avoid any out-of-frame issues, followed by a hemagglutinin-tagged, CHIKV capsid sequence (nt 7541–8323), or SINV TE12 capsid sequence (NCBI accession: NC_001547.1; nt 7647–8630). Site-directed mutagenesis was performed to generate the SSKO viruses or mutated dual luciferase plasmids with PrimeSTAR HS DNA polymerase (Takara Bio USA Inc., San Jose, CA, USA), following the manufacturer’s instructions, and transformed into homemade DH5α competent cells as described above.

### *In vitro* transcription/translation and dual luciferase assay

To evaluate translational efficiency and frameshifting dynamics, dual luciferase plasmids were incubated with the T7 TnT Quick Coupled Transcription/Translation System (Promega, Madison, WI, USA) according to the manufacturer’s protocol, with a 90-minute incubation. A negative control reaction lacking plasmid DNA was included, and all samples and controls were performed in triplicate. The reaction products were subsequently analyzed using a dual luciferase assay.

For the assay, 10 µL of translated products were combined with 50 µL of LARII (Promega) and incubated for 10 minutes before being transferred to a 96-well plate. Luminescence readings were taken with a BioTek Synergy LX Multimode Reader (Agilent) set for a 2-second pre-read delay followed by a 10-second measurement. Next, 50 µL of Stop and Glo Reagent (Promega) was added to each sample, and a second luminescence reading was taken.

Frameshifting ratios were calculated by first subtracting the raw firefly and renilla luciferase values from those of the mock controls, and the ratio was recorded using the formula: [(firefly RLU – mock firefly RLU)/(renilla RLU – mock renilla RLU)] 100. The resulting firefly-to-renilla ratio was then normalized to the corresponding ratio from the respective firefly in-frame control. All constructs were analyzed relative to their respective in-frame controls. Results represent the mean ± SEM from three biological replicates per sample.

### Transfection with dual luciferase plasmids

For cellular experiments, low-passage (<20 passages) HEK293T cells were seeded at ~40% confluency in six-well plates (Greiner Bio-One CellStar) and incubated overnight at 37°C. Midiprepped dual luciferase plasmids (Zymo Research) were prepared for transfection by combining 3,000 ng of DNA with 6 µL of FuGENE 4K Transfection Reagent (Fugene LLC) in 250 µL of Opti-MEM I reduced serum medium (Gibco). After a 15-minute incubation at room temperature, the transfection complexes were added dropwise to the cells in fresh 1× MEM. Cells were incubated at 37°C for 48 hours before harvesting.

At 48 hours post-transfection, cells were washed with 1× PBS and lysed using 1× passive lysis buffer (Promega). Lysates were rocked for 15 minutes at room temperature, transferred to 1.5 mL Eppendorf tubes, and clarified by centrifugation at 14,000 × *g* for 10 minutes. The supernatant was either stored at −20°C or immediately used for the dual luciferase assay exactly as described.

### Quantitative immunoprecipitation

HEK293T cells were transfected with the indicated HA-tagged constructs and incubated for 48 hours. Following incubation, cells were washed with 1× PBS, and a minimal volume of 1× PBS was added to the plates. Cells were irradiated with 5,700 × 100 μJ/cm^2^ in a Stratalinker to cross-link RNA-protein complexes. The cross-linked complexes were solubilized in RIPA buffer (50 mM Tris [pH 7.6], 150 mM NaCl, 1.0% NP-40, 0.5% sodium deoxycholate, and 0.1% SDS) and lysed on ice for 7 minutes. Lysates were vortexed and clarified by centrifugation at 16,000 × *g* for 10 minutes at 4°C. The clarified supernatants were transferred to fresh tubes. The cross-linked lysates were pre-cleared by incubating with 30 µL of 50% slurry Protein A Agarose Beads (9863S, Cell Signaling) for 1 hour at 4°C under constant agitation. The tubes were spun down at 5,000 × *g* for 5 minutes, and the pre-cleared lysates were transferred to fresh tubes. A total of 10% of the sample volume was saved as input reference and stored at −80°C.

For immunoprecipitation, Pierce Anti-HA magnetic beads were washed in 0.05% TBS-T (Thermo Fisher) and incubated with lysates at 4°C overnight with constant agitation. Immunoprecipitated RNA-protein complexes were purified by magnetic separation of the beads. The beads were washed three times with RIPA buffer, followed by two washes with sterile 1× PBS. The beads were resuspended in 1× PBS, and 10% of the total volume was saved for western blot analysis. The remaining beads were used for RNA analysis as described below.

To verify the IP was specific to HA-tagged capsid, beads were boiled in 6× SDS dye and subjected to western blot analysis alongside the corresponding input and unbound lysate samples. For RNA analysis, the purified bead-bound RNA-protein complexes and input samples were treated with Proteinase K and RNAse Inhibitor Murine at 55°C for 30 minutes with gentle agitation to release RNA fragments. RNA was extracted using the Aurum Total RNA Mini Kit (Bio-Rad Laboratories) and resuspended in 20 µL elution buffer. cDNA was synthesized, and qRT-PCR was performed as described in the specific infectivity section with primers targeting the 9900 region. RNA-IP values were normalized first to GAPDH and then to corresponding input samples to control for differences in starting material. Normalized values were made relative to WT HA-capsid. All samples were collected and analyzed in biological triplicate.

### Western blotting

Five micrograms of whole cell lysates from the immunoprecipitation protocol was separated by 10% SDS-PAGE (Bio-Rad Laboratories, Hercules, CA, USA) and run at 150 V for 1 hour. The gel was transferred to a polyvinylidene difluoride (PVDF) membrane and blocked in 5% TBSTM (Tris buffered saline 1×, 5% dry milk, 0.1% Tween-20) for 1 hour at room temperature. Following blocking, the membrane was incubated overnight with rabbit anti-HA tag polyclonal antibody (C29F4) and rabbit anti-beta-actin (#4967) antibody (both from Cell Signaling Technology) at 4°C in 2.5% TBSTM. The membrane was washed three times with TBST before incubation with goat anti-rabbit AlexaFluor 750 (#A-21039 ThermoFisher, Waltham, MA, USA) diluted with 2.5% TBSTM for 1 hour at room temperature in the dark. Following a minimum of three washes with TBST, the membrane was imaged using a Bio-Rad ChemiDoc MP Imaging System. All western blot experiments were repeated at least three times independently with similar results. Representative images are shown.

### JAK-STAT inhibition with ruxolitinib

HEK293T cells were seeded in 24-well plates and allowed to adhere for 24 hours. The medium was then removed and replaced with fresh 1× MEM. Pre-treatment of half of the wells was done by adding either 0.1% DMSO or 300 nM ruxolitinib (#83405, Cell Signaling). After 24 hours of pre-treatment, the medium was removed, and cells were infected at 0.1 PFU/cell with either mKate-tagged CHIKV or 9900 viruses. Following the infection period, virus-containing medium was discarded, cells were washed with 1× PBS, and fresh 1× medium containing 0.1% DMSO or 300 nM ruxolitinib was added to the cells that received prior treatment. Remaining wells received 1× MEM.

At 48 HPI, the cell culture medium was harvested and spun down, and the supernatant was titered on BHK-21 cells. The HEK293T cells were lysed, RNA was extracted, and cDNA synthesis followed by qRT-PCR was performed as above with minor modifications. The primers used for qRT-PCR were GAPDH and ISG15, and relative expression was calculated using the delta-delta Ct method, with GAPDH as the reference control. All data were obtained from biological triplicates, and qRT-PCR was performed in technical duplicates.

### Multiple sequence alignment

All available CHIKV sequences with complete nucleotide profiles and full virus sequence length (>11,000 bases) were downloaded from the NCBI Virus database (ncbi.nlm.nih.gov/labs/virus/vssi; taxid: 37124). These sequences were aligned using SnapGene with MAFFT, and the nucleotide region corresponding to positions 9869–9913 (the 9900 region) was extracted from the alignment. The extracted sequences were then used as input for WebLogo (https://weblogo.berkeley.edu/logo.cgi) to visualize nucleotide variation across strains. This analysis was used to determine whether the mutations introduced at the 9900 region are present in any naturally occurring CHIKV sequences.

### Statistical analyses

All statistical analyses were performed using GraphPad Prism 9 (GraphPad Software Inc., San Diego, CA, USA). Data reflect a minimum of three biological replicates per sample in each assay. Dual luciferase data are reflective of the guidelines highlighted in references 35, 49.

## Data Availability

All data generated in this study will be made available upon request.

## SUPPLEMENTAL MATERIAL

Supplemental figures jvi.01393-25-s0001.pdf Figures S1 to S3.

ASM does not own the copyrights to Supplemental Material that may be linked to, or accessed through, an article. The authors have granted ASM a non-exclusive, world-wide license to publish the Supplemental Material files. Please contact the corresponding author directly for reuse.
