# Supplementary material for: The role of chikungunya virus capsid-viral RNA interactions in programmed ribosomal frameshifting
Source: J Virol. 2025 Oct 13;99(11):e01393-25. doi: 10.1128/jvi.01393-25 (PMC12645981; doi:10.1128/jvi.01393-25)
Supplement: Supplemental figures — Figures S1 to S3. [file jvi.01393-25-s0001.pdf]

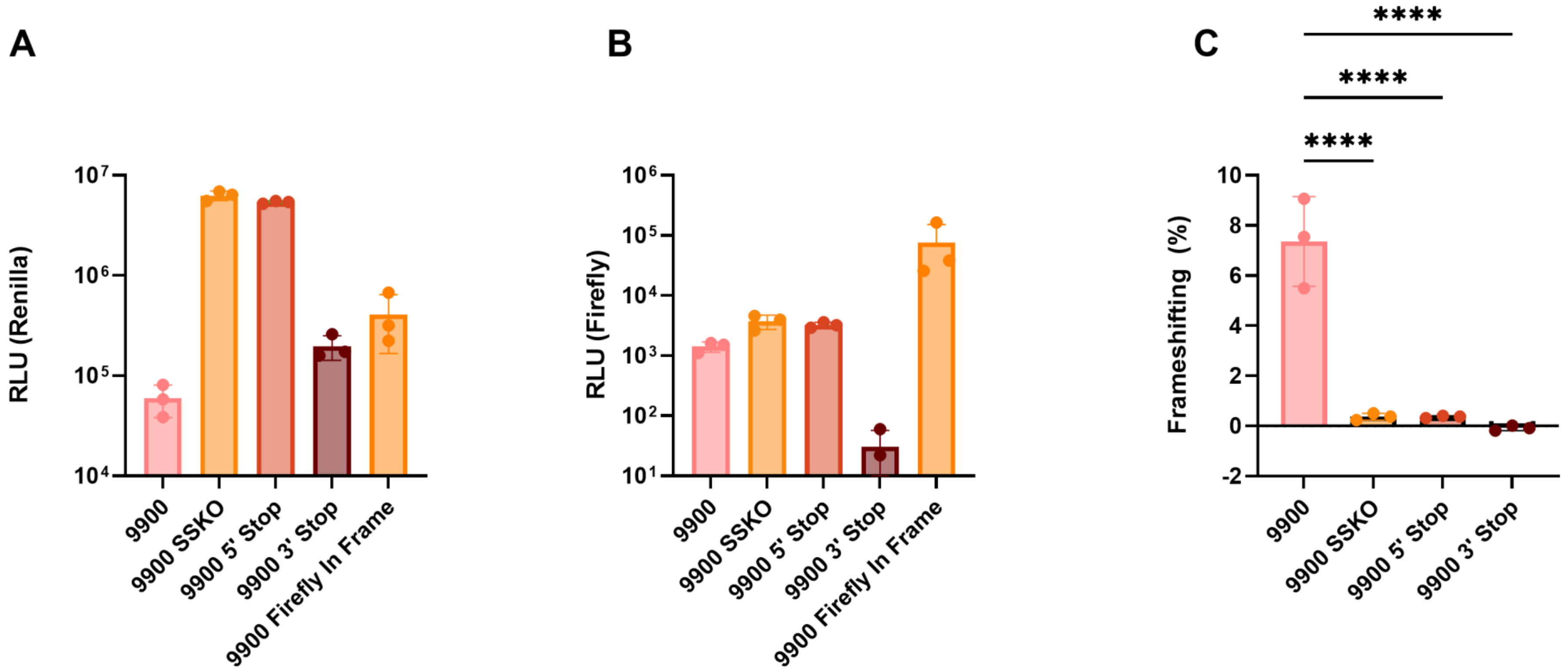

Supplemental Figure 1. 9900 control constructs undergo -1 PRF similarly to WT controls in a minimal translational environment. (*A*) Renilla luciferase activity is presented as a measure of translation occurring in the 0-frame. Mock showed minimal Renilla background (~10^3 RLU). The Firefly In Frame Construct is in the 9900 background. (*B*) Firefly luciferase activity reflects translation via -1 PRF. As expected, the mock control demonstrated minimal Firefly activity (~10^2 RLU). (*C)* Relative frameshifting efficiency (%) was calculated as the ratio of firefly luciferase to renilla luciferase, normalized to the Firefly In Frame control. The control constructs in a 9900 background yielded similar results to WT controls. Statistical significance was assessed by one-way ANOVA with Tukey's post hoc test to correct for multiple comparisons relative to 9900 (****$p < 0.0001$). Data are shown as mean ± SD of biological replicates.

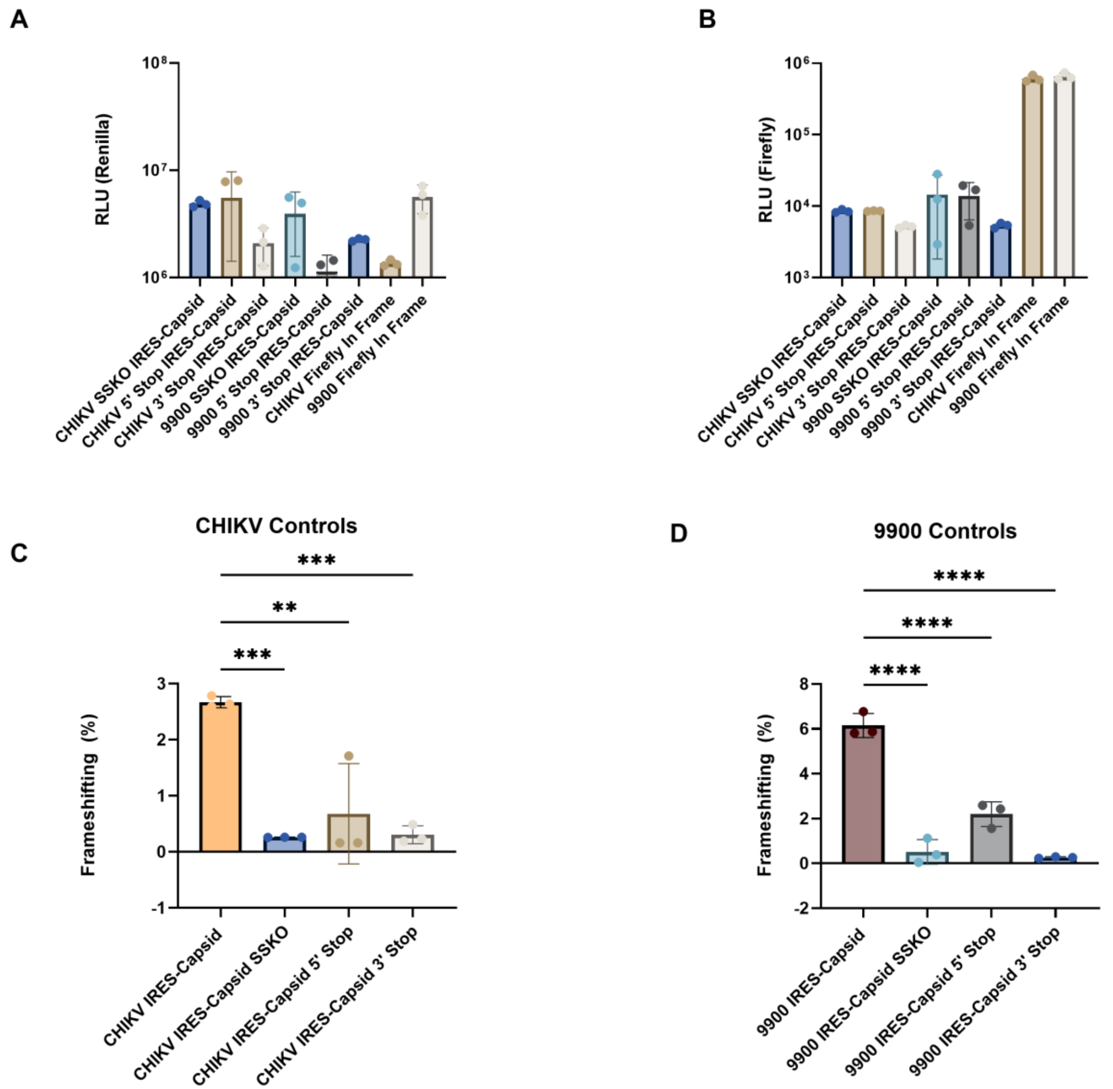

Supplemental Figure 2. Capsid-containing dual luciferase reporters indicate that capsid expression does not significantly influence aberrant PRF. (*A*) Renilla luciferase activity serves as a measure of translation in the 0-frame. Mock exhibited low background Renilla activity (~10^4 RLU). (*B*) Firefly luciferase activity reflects translation occurring via -1 PRF. Mock exhibited low background Firefly activity (~10^3 RLU). (*C-D*) Relative frameshifting efficiency is calculated as the ratio of Firefly to Renilla luciferase activities for the indicated constructs. CHIKV control constructs (*C*) and 9900 control constructs (*D*) are shown, normalized to the WT IRES-Capsid or 9900 IRES-Capsid Firefly In-Frame controls. Data represent the mean ± SD of biological replicates, performed in triplicate. Statistical significance was determined using one-way ANOVA with Tukey's post hoc test to correct for multiple comparisons relative to the CHIKV or 9900 IRES-Capsid construct, respectively (**$p < 0.01$. ***$p < 0.001$. ****$p < 0.0001$).

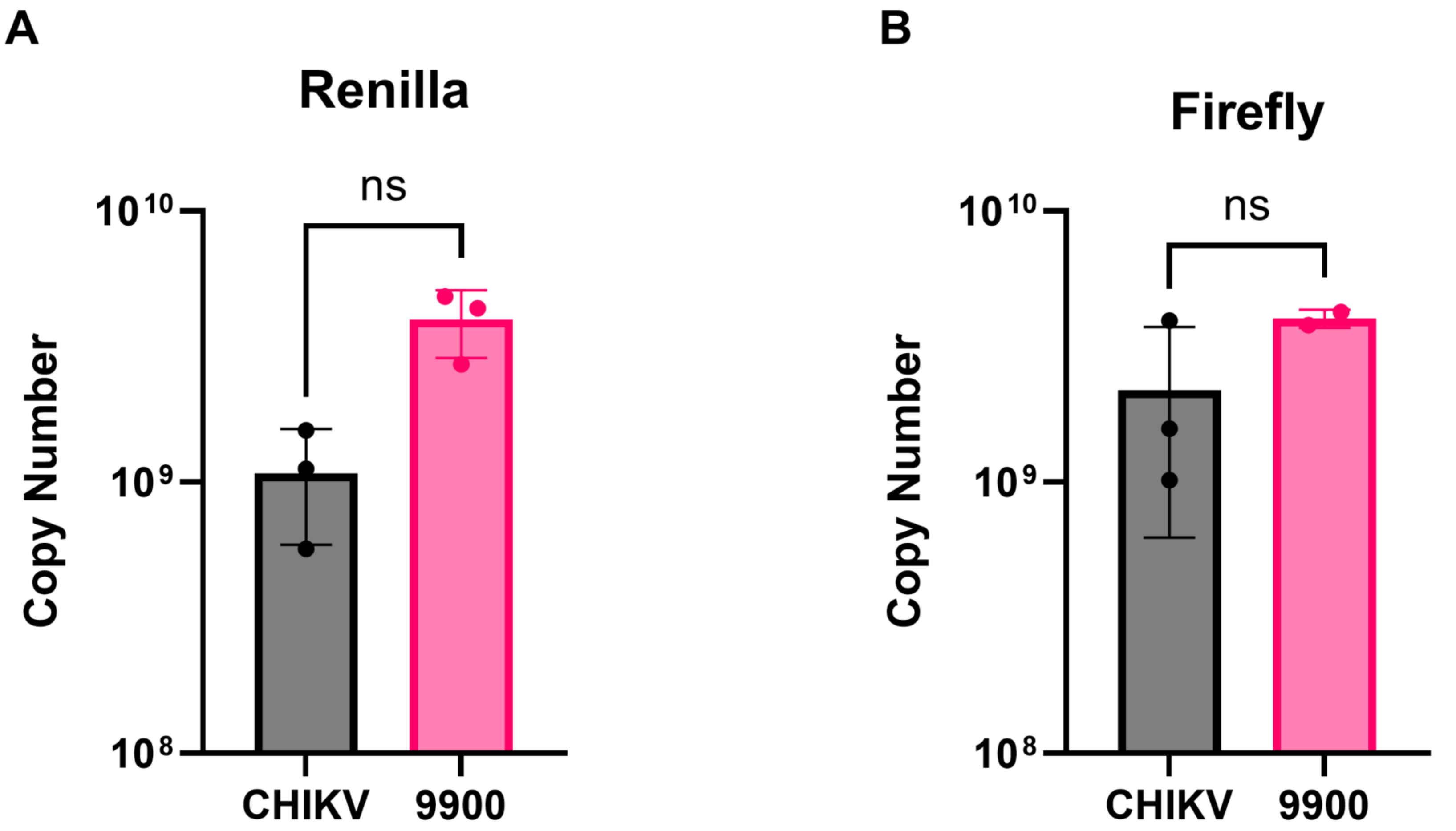

Supplemental Figure 3. Equivalent expression of dual luciferase construct-derived RNAs from CHIKV and 9900. HEK293T cells were transfected in triplicate with IRES-capsid dual luciferase constructs containing either the CHIKV or 9900 6K/TF sequence. At 48 hours post-transfection, RNA was harvested from cells, and Renilla (*A*) and Firefly (*B*) luciferase transcript copy numbers per 1 mL were quantified by qRT-PCR. Absolute quantification was performed using standard curves generated from serial dilutions of the corresponding plasmids. Each qRT-PCR reaction was performed in technical duplicate. No significant differences (ns) were observed between CHIKV and 9900 constructs, confirming equivalent expression of both reporter transcripts. Statistical significance was determined by the p-values from multiple paired t-tests with a Bonferroni correction where necessary.
